# Energy Consumption, Carbon Emissions and Global Warming Potential of Wolfberry Production in Jingtai Oasis, Gansu Province, China

**DOI:** 10.1007/s00267-019-01225-z

**Published:** 2019-11-20

**Authors:** Yaolin Wang, Quanlin Ma, Yingke Li, Tao Sun, Hujia Jin, Chuanyan Zhao, Eleanor Milne, Mark Easter, Keith Paustian, Hoi Wen Au Yong, John McDonagh

**Affiliations:** 1 State Key Laboratory of Desertification and Aeolian Sand Disaster Combating, Gansu Desert Control Research Institute, Lanzhou, China; 2 Tianshui Normal University, Tianshui, China; 3 State Key Laboratory of Grassland and Agro-Ecosystems, School of Pastoral Agriculture, Lanzhou University, Lanzhou, 730000 China; 4 The Natural Resource Ecology Laboratory, Colorado State University, Fort Collins, CO 80523-1499 USA; 5 School of International Development, University of East Anglia, Norwich, NR4 7TJ UK

**Keywords:** Energy use, Greenhouse gas emissions, Global warming potential, Wolfberry plantation

## Abstract

During the last decade, China's agro-food production has increased rapidly and been accompanied by the challenge of increasing greenhouse gas (GHG) emissions and other environmental pollutants from fertilizers, pesticides, and intensive energy use. Understanding the energy use and environmental impacts of crop production will help identify environmentally damaging hotspots of agro-production, allowing environmental impacts to be assessed and crop management strategies optimized. Conventional farming has been widely employed in wolfberry (*Lycium barbarum*) cultivation in China, which is an important cash tree crop not only for the rural economy but also from an ecological standpoint. Energy use and global warming potential (GWP) were investigated in a wolfberry production system in the Yellow River irrigated Jingtai region of Gansu. In total, 52 household farms were randomly selected to conduct the investigation using questionnaires. Total energy input and output were 321,800.73 and 166,888.80 MJ ha^−1^, respectively, in the production system. The highest share of energy inputs was found to be electricity consumption for lifting irrigation water, accounting for 68.52%, followed by chemical fertilizer application (11.37%). Energy use efficiency was 0.52 when considering both fruit and pruned wood. Nonrenewable energy use (88.52%) was far larger than the renewable energy input. The share of GWP of different inputs were 64.52% electricity, 27.72% nitrogen (N) fertilizer, 5.07% phosphate, 2.32% diesel, and 0.37% potassium, respectively. The highest share was related to electricity consumption for irrigation, followed by N fertilizer use. Total GWP in the wolfberry planting system was 26,018.64 kg CO_2_ eq ha^−1^ and the share of CO_2_, N_2_O, and CH_4_ were 99.47%, 0.48%, and negligible respectively with CO_2_ being dominant. Pathways for reducing energy use and GHG emission mitigation include: conversion to low carbon farming to establish a sustainable and cleaner production system with options of raising water use efficiency by adopting a seasonal gradient water pricing system and advanced irrigation techniques; reducing synthetic fertilizer use; and policy support: smallholder farmland transfer (concentration) for scale production, credit (small- and low-interest credit) and tax breaks.

## Introduction

Global greenhouse gas (GHG) emissions from food production nearly doubled during the period between 1961 and 2011 (FAOSTAT 2014), and will continue to rise as global crop demand is projected to have a 100–110% increase between 2005 and 2050 (Tilman et al. 2011). This alarming increase is closely correlated with intensive energy use. Agriculture is one of the major energy consumers and has experienced rapid intensification in recent decades (Nemecek et al. 2011). The production, transportation, processing, etc. of the agro-food sector contributes ~20% to global anthropogenic GHG emissions (FAO 2012). Notably, emissions from agricultural production account for over 80–86% of the global total food system emissions (Vermeulen et al. 2012). Recent studies have suggested that the agro-food sector is a significant contributor to global warming (Beccali et al. 2009; Michos et al. 2012).

As the largest food producer and consumer in the world, China has been one of the largest anthropogenic GHG emitters and currently emits around 20% of global GHGs (Leggett et al. 2011). Agricultural GHG emissions have been estimated at 11% of China’s national emissions, growing rapidly from 605 Mt CO_2_ eq in 1994 to 820 Mt CO_2_ eq in 2005 with a mean annual growth rate of 2.8% (Nayak et al. 2015; Lin et al. 2015). China is also the largest chemical fertilizer consumer with a N_2_O emissions increase from 0.18 Tg in 1978 to 0.41 Tg in 2010 (Cui et al. 2013).

The Chinese government made a commitment at the 2009 U.N. Climate Change Conference in Copenhagen that, by 2020, China’s CO_2_ emissions will drop with a target of 40–45% above the emission level in 2005 (Yang and Chen 2013). Agriculture is among the major sectors earmarked to reduce energy use while low carbon approaches in crop production is part of China's national climate change mitigation strategy. Accordingly, Gansu province has been designated as a circular economic demonstration area in China and low carbon and organic farming initiatives are a key area to attain green growth (Deng 2014).

Cash tree production has increased rapidly in China over the last decade, making it one of the largest fruit producers in the world (Su 2012; Cerutti et al. 2014). Cash tree production is an intensive agricultural system with high inputs of fertilizers, pesticides, irrigation, fossil fuels, and other materials (Li et al. 2010). However, growers are generally motivated by the notion of "the more fertilizer and irrigation, the higher the yield output," instead of energy efficiency and judicious management, with extensive management as a result, causing environmental issues (Cao 2014; Jiao et al. 2016). Efficient energy use in agriculture would minimize environmental burdens, decrease reliance on nonrenewable energy, and form a sustainable and economical production system (Uhlin 1998). In recent years, many studies have been conducted to determine the energy use pattern and efficiency of cash tree production; for example apple production in Greece (Strapatsa et al. 2006), energy inputs, outputs, and GHG emissions in organic, integrated and conventional peach orchards (Michos et al. 2012), resource consumption and emissions in olive oil production (Avraamides and Fatta 2008), environmental impacts in citrus production (Dwivedi et al. 2012) and energy use and GHG emissions in almond production in the United States (Kendall et al. 2015). Liu et al (2010a) compared carbon footprints of organic and conventional pear planting in northern China using life cycle analysis and indicated options available to reduce energy use and carbon emissions. In addition, Wang et al (2015) assessed the impact of diversified management practices of winter wheat on total GHG emissions.

Wolfberry (*Lycium barbarum L*.), is a shrub with its fruits being served as tonic food and traditional Chinese medicine, sold not only in domestic market but also exported to other countries and regions with good and stable prices (Li et al. 2017). It is salt tolerant, drought resistant, fast-growing, and fruits in the first year of planting. It is widely used for saline land improvement and rural economic development. Thus the area under wolfberry cultivation has expanded in northern China over the last few decades. However, there is little information on energy use efficiency and global warming potential (GWP) in wolfberry production systems in China.

Therefore, a combination of energy input and environmental impact analysis in a production system is necessary to optimize crop management practices, reduce the environmental impacts and promote sustainable development (Ming et al. 2015). The objectives of this study were to: (i) analyze the output–input energy; (ii) calculate total GHG emissions (CO_2_, N_2_O, and CH_4_), and (iii) determine GWP per unit of chemical input and output in a wolfberry production system in Gansu, with the aim of identifying possible pathways to reducing energy consumption and mitigating environmental impacts in cash tree crop production.

## Materials and Methods

The study was conducted in wolfberry plantations in the full bearing period in the irrigated area of Jingtai County (103°33′–104°43′ E, 36°43′–37°28′ N) in northern Gansu Province, northwest China in 2013–2014. Jingtai County is one of the main wolfberry producers in Gansu. The region has a dry continental climate with an average annual temperature of about 8.6 °C, a maximum temperature of 38.6 °C in July and a minimum temperature of −27.3 °C in January. Annual rainfall is ~180 mm, of which 90% falls between April and September.

The region’s agriculture strongly depends on irrigation by an electrically powered water lifting project from the Yellow River with a total lift of 713 m. Wolfberry cultivation is managed on a household farm basis. Most of households in the irrigated region are engaged mainly in wolfberry cultivation and the region is the origin of wolfberry cultivation in Gansu. The plantation size across households ranges from 0.3 to 3 ha and the planting density is 5250 trees ha^−1^. The main field management activities are given in Table 1. Wolfberry growing has stimulated processing, trade and job opportunities, becoming a pillar of the local economy (Zhang et al. 2010). Meanwhile, based on wolfberry planting a new ecological agriculture model is taking shape, namely, free range chicken production within the plantation (Sheng and Su 2011).

**Table 1:** The main field management activities involved in wolfberry planting in Jingtai, Gansu, China

Field operations	Time	Brief frequency or intensity description
Fertilizer application	Beginning of March	Apply sheep manure^a^ (*N* = 0.65%, P_2_O_5_ = 0.47%, K_2_O = 0.21%) by spade
	Beginning of March, beginning of May, be gaining of June, middle of July.	Apply chemical fertilizers by spade
	End of May, end of June, middle of July	Spray KH_2_PO_4_ with tricycle driven sprayer
Pruning (winter, spring, and summer)	Beginning of December to end of March, middle to end of May, and end of May to end of June	Pruning with special scissors with heavy winter pruning
Weeding	Before middle of May	By tiller rotary
	After middle of May	Spray herbicides by sprayer manually
Irrigation	After end of April to end of October	8 times per year
Pest management	Growing season	Spray chemical pesticides 6 times with tricycle driven sprayer
Harvesting	Middle of June to beginning of September	By hand
Fruit air drying	Harvesting season	By hand

^a^He et al. 2011

The investigation was carried out in 52 household farms, selected with the simple random sampling method (Fan et al. 2016) in Jingtai’s wolfberry planting region. Data on farm practices, inputs, and consumption of resources at each stage of the production chain were collected with a household survey questionnaire via face–face interviews. In addition, information was also collected from local Forestry Bureau, Forestry and Agricultural Technical Extension Stations and Agricultural Machinery Service.

The fruit yield and pruning wood were designated as the energy output. The energy inputs included human labor, machinery, diesel fuel, chemical fertilizers, pesticides, electricity, and irrigation water. Input energy in wolfberry production systems can be divided into direct, indirect, and renewable and nonrenewable energies. Direct energy in the study system involved human labor, diesel fuel, water for irrigation, and electricity. Indirect energy included chemical fertilizer, manure, pesticide, machinery, and tools. Also, renewable energy resources were human labor, water for irrigation, and manure and nonrenewable energy resources were electricity, chemical fertilizer, diesel fuel, pesticide, machinery, and tools.

All of the inputs and outputs were converted into energy equivalents by multiplying the quantity of inputs by their corresponding energy coefficients. The energy equivalents of inputs used in this study are given in Table 2. The energy efficiency of wolfberry production was evaluated based on the input–output analysis. For the estimation of fossil energy used in wolfberry planting, both direct (fossil energy consumed on the farm) and indirect energy (fossil energy for production of synthetic fertilizers, chemical pesticides, machinery, etc.), were considered. In addition, the energy input of human labor was considered. The energy equivalent of water for irrigation input means indirect energy of irrigation consisting of the energy consumed for manufacturing the materials for the dams, canals, pipes, pumps, and equipment as well as the energy for constructing the works and building the on-farm irrigation systems (Khan et al. 2009). Embodied energy in machinery was expressed in terms of MJ kg^−1^. To analyze embodied energy in the production of farm machinery, it was assumed that energy is depreciated during the economic lifetime of the machinery (Iriarte et al. 2010); Eq. (1) was used to calculate the weight of machinery depreciated per hectare of wolfberry production during the production period (Mousaviavval et al. 2011):

1 $${TW} = {G} \times {W}_{h}{/T}$$

where *TW* denotes the depreciated machinery weight (kg ha^−1^); *G* refers to the total machine weight (kg); *W*_*h*_ stands for the time of machine use per unit area (h ha^−1^) and *T* is the economic lifetime of machine (h).

**Table 2:** Energy equivalents of inputs and outputs

Inputs and output	Unit	Energy equivalent (MJ unit^−1^)	Mass (kg)	Life (years)	Reference
A. Inputs					
1. Human labor	h	1.95			(Taylor et al. 1993)
2. Machinery	kg	210.00		10.00	(Liu et al. 2010a)
(a) Sprayer			7.00		
(b) Rotary tiller			70.00		
(c) Agricultural tricycle			1120.00		
3. Diesel fuel	L	47.79			(Cervinka 1980)
4. Chemical fertilizer					(Yin et al. 1998)
(a) Nitrogen (N)	kg	50.00			
(b) Phosphate (P_2_O_5_)	kg	12.00			
(c) Potassium (K_2_O)	kg	4.22			
5. Pesticides					
(a) Herbicides	kg	288.00			(Liu et al. 2010a)
(c) Pesticides	kg	237.00			(Liu et al. 2010a)
6. Farmyard manure	kg	0.30			(Kizilaslan 2009)
7. Electricity	kW h	12.50			(Liu et al. 2010a)
8. Water for irrigation	m^3^	1.02			(Rajaeifar et al. 2014)
9. Tools (scissors, hoes, spades, etc.)	h	0.10			(Liu et al. 2010a)
B. Output					
(a) Yield	kg	18.36			(Xu et al. 2007)
(b) Prunings	kg	18.48			(Liu 2011)

Energy use efficiency, energy productivity, net energy, and specific energy were determined according to Eqs. (2–5), respectively, (Asgharipour et al. 2012):

2 $$\begin{array}{l}{\mathrm{Energy}}\,{\mathrm{use}}\,{\mathrm{efficiency = Energy}}\,{\mathrm{output}}\\ \,\left( {{\mathrm{MJ}}\,{\mathrm{ha}}^{{\mathrm{ - 1}}}} \right) {\mathrm{/Energy}}\,{\mathrm{input}}\,\left( {{\mathrm{MJ}}\,{\mathrm{ha}}^{{\mathrm{ - 1}}}} \right)\end{array}$$

3 $$\begin{array}{l}{\mathrm{Energy}}\,{\mathrm{productivity = Crop}}\,{\mathrm{output}}\,\left( {{\mathrm{kg}}\,{\mathrm{ha}}^{{\mathrm{ - 1}}}} \right){\mathrm{/}}\\ {\mathrm{Energy}}\,{\mathrm{input}}\,\left( {{\mathrm{MJ}}\,{\mathrm{ha}}^{{\mathrm{ - 1}}}} \right)\end{array}$$

4 $$\begin{array}{l}{\mathrm{Net}}\,{\mathrm{energy = Energy}}\,{\mathrm{output}}\,\left( {{\mathrm{MJ}}\,{\mathrm{ha}}^{{\mathrm{ - 1}}}} \right)\\ {\mathrm{-}} {\mathrm{Energy}}\,{\mathrm{input}}\,\left( {{\mathrm{MJ}}\,{\mathrm{ha}}^{{\mathrm{ - 1}}}} \right)\end{array}$$

5 $${\mathrm{Specific}}\,{\mathrm{energy = Energy}}\,{\mathrm{input}}\,\left( {{\mathrm{MJ}}\,{\mathrm{ha}}^{{\mathrm{ - 1}}}} \right){\mathrm{/Yield}}\,\left( {{\mathrm{kg}}\,{\mathrm{ha}}^{{\mathrm{ - 1}}}} \right)$$

The amount of GHG emissions from chemical inputs in wolfberry production per hectare were calculated by using CO_2_, N_2_O, and CH_4_ emissions coefficients of chemical inputs that are shown in Table 3. GHG emissions can be calculated and expressed in per unit land area, per unit crop produce, per unit energy input or output, and per unit economic output. In this study, the direct emissions from GHGs resulting from chemical inputs were calculated per unit cropland area. Each GHG, i.e., carbon dioxide (CO_2_), methane (CH_4_), and nitrous oxide (N_2_O) has a GWP, which is the warming influence relative to that of carbon dioxide. The emissions are measured in terms of a reference gas, CO_2_ (IPCC et al. 1995). The GWPs of CO_2_, CH_4_, andN_2_O (with a time span of 100 years) are 1, 25, and 298, respectively. The total GHG effect related to emissions of GHGs is determined as follows (Kramer et al. 1999):

6 $${\mathrm{Greenhouse}}\,{\mathrm{effect = }}{\Sigma} {{\mathrm{GWP}}_{\mathrm{i}} \times {\mathrm{m}}_{\mathrm{i}}}$$

where m_i_ is the mass (in kg) of the emission gas. The score is expressed in terms of CO_2_ equivalents.

**Table 3:** Gaseous emissions (g) per unit of chemical sources and their global warming potential (GWP)

Inputs	CO_2_	N_2_O	CH_4_	Reference
1. Diesel (L)	3875.70	0.14	0.65	Yang et al. (2014)
2. Nitrogen fertilizer (kg)	10,125.56	0.17	0.24	Yang et al. (2014)
3. Phosphate (P_2_O_5_) (kg)	1496.49	0.02	0.02	Yang et al. (2014)
4. Potassium (K_2_O) (kg)	973.20	0.03	0.04	Yang et al. (2014)
5. Electricity (kW h)	948.48	0.01	0.01	Yang et al. (2014)
GWP CO_2_ equivalence factor	1.00	298.00	25.00	Yang et al. (2014)

## Results and Discussion

### Energy Input–Output Analysis in Wolfberry Production

Energy inputs and outputs in the wolfberry production system, their energy equivalents, and percentages in the total energy input are given in Table 4. The total energy input for wolfberry production was 321,800.7 MJ ha^−1^. The highest share in the total energy input was found to be electricity consumption for lifting water for irrigation accounting for 68.5%, followed by chemical fertilizers use 11.37%, chemicals 5.45%, and human labor 5.16%, respectively. The wolfberry production system is characterized by high energy inputs in electricity use, fertilizer application, in particular nitrogen fertilizer. Beigi et al. (2015) reported the highest share of electricity (58%) consumed for pumping water for irrigation in almond production in arid Tokan Province, Iran. Tabatabaie et al. (2012, 2013) showed a similar trend in plum (80%) and pear (78%) production in arid areas in Iran. High electricity consumption for water lifting from the Yellow River, with a high lift, is a salient feature of wolfberry planting in Jingtai region, caused by extravagant water use for irrigation due to poor irrigation efficiency as well as rigid water pricing, a policy based mechanism and a result of the planned economy. Current water prices are too low, about 51.1% of the cost price (Peng 2011). A water consumption of 12,600 m^3^ ha^−1^ for irrigation has been adopted by growers in most cases so far, far beyond the irrigation water norm of 5550–6270 m^3^ ha^−1^ for wolfberry (Zhang et al. 2010; Zeng et al. 2013), causing serious waste of water, second salinization of soil, high energy inputs, and increased GHG emissions, which has consequently led to a reduced sustainability of the production system.

**Table 4:** Energy inputs, outputs, and the ratio in wolfberry production systems

Inputs and output (unit)	Quantity per unit area (ha)	Total energy equivalents	%
A. Inputs			
1. Human labor (h)	8520.00	16,614.00	5.16
2. Machinery (kg)	2915.50	2915.50	0.91
Sprayer		0	0
Rotary tiller		0	0
Agricultural tricycle		0	0
3. Diesel fuel (L)	147.00	7025.13	2.18
4. Chemical fertilizer (kg)			
Nitrogen (N)	546.00	27,300.00	8.48
Phosphate (P_2_O_5_)	760.20	9122.40	2.83
Potassium (K_2_O)	45.90	193.70	0.06
5. Farmyard manure (kg)	24,970.0	7491.00	2.33
6. Chemicals (kg)			
Pesticides	63.00	14,931.00	4.64
Herbicides	9	2592.00	0.81
7. Electricity (kW h)	17,640.0	220,500.00	68.52
8. Water for irrigation (M^3^)	12,600.00	12,852.00	3.99
9. Tools (scissors, hoes, spades, etc.)	2640.00	264.00	0.08
Total input energy		321,800.73	100
B. Output			
Yield (kg)	4500.00	82,620.00	
Prunings (kg)	4560.00	84,268.80	
Total output energy		166,888.80	

Of the fertilizer energy input, the share of nitrogen fertilizer was the highest (8.48%), incurred by heavy use and high embodied energy intensity; phosphate the second (2.83%), and potassium the third (0.06%). Nitrogen application makes up the highest share in the fertilizers energy input in apricot production in Turkey (Esengun et al. 2007). Similar trends have also been reported for pistachio, orange, and peach production respectively (Külekci and Aksoy 2013; Ozkan et al. 2004; Ghatrehsamani et al. 2016).

In terms of the chemicals energy input, the share of pesticides use was the highest (4.64%) and herbicides input the second (0.81%). A higher share of pesticides in the total input energy is also found in peach production system in Turkey (Yildiz et al. 2016).

Human labor, a renewable source of energy, was in the fourth place. Both fruit harvest and pruning consist of the bulk of the labor energy input with fruit harvest accounting for 60% and pruning for 13%, respectively, in wolfberry production systems in Jingtai region (Wang et al. 2015). The highest use of human labor is also found in harvesting (56%) and pruning operations (23%) in apple production in Iran (Rafiee et al 2010) as well as in fruit harvest (46%) in walnut production systems in Turkey (Gundogmus 2013).

The wolfberry fruit yield was 4500 kg ha^−1^ on average and total brushwood pruned was 4560 kg ha^−1^ in the production system. Accordingly, their energy equivalents were 82,620 and 84,268.8 MJ ha^−1^, respectively. Total energy output was calculated for both fruit and trimmings energy equivalents. Pruning is an important part of a wolfberry production system with a view to gaining a stable and high yield. Pruned wood is a byproduct of wolfberry planting, used as farm household fuel wood in the wolfberry planting area.

### Energy Use Indicator Analysis in Wolfberry Production Systems

Results of energy indicators for wolfberry production are given in Table 5. Consumed and produced energy intensities were 32.21 and 16.71 MJ m^−2^, respectively. Energy use efficiency was 0.26 considering fruits only, and 0.52 taking into account both fruits and pruned wood, indicating that 0.52 energy units were obtained per unit of energy input in the wolfberry production system. Energy use efficiency for organic wolfberry is 1.4 in Aksaray Province of Turkey (Oğuz et al. 2018). Energy ratios of other agricultural products, such as 1.16 for apple (Rafiee et al. 2010), 0.87 for orange (Mohammadshirazi et al. 2012), 0.62 for almond (Beigi et al. 2015), 0.69 for conventional pear production (Liu et al. 2010a), and 0.46 for organic pear (Liu et al. 2010b), have been reported. Hetz (1998) reported that the energy ratio of fruit production ranged between 0.44 and 2.22 in Chile. Low energy use efficiency in the wolfberry planting system resulted from high energy inputs such as electricity consumption, chemical fertilizers, and biocides use. Results show that energy productivity in the wolfberry production system in Jingtai region was 0.014 kg MJ^−1^. Energy productivities of other crops have been revealed as 0.42 kg MJ^−1^ for apple (Strapatsa et al. 2006), 0.43 kg MJ^−1^ for tangerine (Mohammadshirazi et al. 2012), 0.08 kg MJ^−1^ for oil olive (Rajaeifar et al. 2014), 0.018–0.025 kg MJ^−1^ for various almond varieties (Torki-Harchegani et al. 2015), 0.656 kg MJ^−1^ for orange, and 0.555 kg MJ^−1^ for lemon (Ozkan et al. 2004). The differences arise from different plants, products, levels of management etc. Net energy was −154,911.9 MJ ha^−1^. A negative value of net energy implies wolfberry production is inefficient in energy use, thus indicating that energy is being lost during wolfberry production. A similar issue is also revealed in almond production systems in arid Chaharmahal-Va-Bakhtiariprovince, Iran (Beigi et al. 2015). Specific energy refers to how much energy is consumed per unit of harvested products. It was 71.51 MJ kg^−1^ for wolfberry production in the Jingtai area. Specific energies are 2.66 MJ kg−1 for organic wolfberry in Turkey (Oğuz et al. 2018), 1.23 MJ kg^−1^ for kiwifruit (Mohammadi et al. 2010), 60.91–110.31 MJ kg^−1^ for almond (Torki-Harchegani et al. 2015), and 12.7 MJ kg^−1^ for oil olive (Rajaeifar et al. 2014) in Iran. By contrast, the specific energy for wolfberry production is higher in the Jingtai region. Total energy input consumed falls into four categories: direct, indirect, renewable, and nonrenewable energy inputs, given in Table 6. The share of direct energy inputs was four times greater than the indirect energy use. And nonrenewable energy use (88.52%) was far larger than the renewable energy input (11.48%). Similar results are found for almond, pear, and cherry (Osman et al. 2018; Kizilaslan 2009). The high portion of nonrenewable and direct energy consumption with poor efficiency means there are serious problems existing in the production systems, not only resulting in environmental impacts, such as pollution, but confronting us with the dilemma of depletion of such invaluable resources. This is, motivated by excessive water use for irrigation induced by irrational water pricing systems, poor irrigation efficiency, and a high rate of chemical use, in particular, excessive fertilizer application.

**Table 5:** Energy indices in wolfberry planting

Indicators	Unit	Quantity
Energy input	MJ ha^−1^	321,800.73
Energy output	MJ ha^−1^	166,888.80
Yield	kg ha^−1^	4500.00
Prunings	kg ha^−1^	4560.00
Consumed energy intensity	MJ m^2^	32.21
Produced energy intensity	MJ m^2^	16.71
Energy use efficiency		0.52
Energy productivity	kg MJ^−1^	0.014
Net energy	MJ ha^−1^	−154,911.93
Specific energy	MJ kg^−1^	71.51

**Table 6:** Total energy input in the form of direct, indirect, renewable, and nonrenewable for wolfberry production

Indicators	Quantity (MJ ha^−1^)	Percentage (%)
Direct energy^a^	256,991.13	79.86
Indirect energy^b^	64,545.60	20.14
Renewable energy^c^	36,957.00	11.48
Nonrenewable energy^d^	284,579.73	88.52
Total energy input	321,800.73	

^a^Includes electricity, human labor, diesel fuel, and water

^b^Includes chemical fertilizer, farmyard manure, chemicals, machinery, and tools

^c^Includes human labor, farmyard manure, and water for irrigation

^d^Includes diesel fuel, electricity, chemicals, chemical fertilizer, machinery, and tools

### GHG Emissions and Global Warming Potential (GWP)

GHG emissions from chemical inputs to the wolfberry cultivation system are given in Table 7. Rates of CO_2_, N_2_O and CH_4_ emissions were 25881.83, 0.42, 0.46 kg ha^−1^, respectively, with CO_2_ making up 99.997%. Wang et al. (2007) revealed a similar pattern in winter wheat-summer maize production system in the North China Plain. In addition, Kramer et al. (1999) found the agricultural products produced 1100 kton CO_2_, 3 kton N_2_O and 0.7 kton CH_4_, respectively, with CO_2_ being dominant in the Netherlands. In terms of CO_2_ emissions, the highest share was related to electricity consumption (64.6%) followed by N fertilizer use (27.7%) while in the case of N_2_O and CH_4_ emissions, both showed a similar trend to CO_2_.

**Table 7:** Gaseous emissions (kg ha^−1^) from chemical sources and their GWP in wolfberry production system

Inputs	CO_2_	N_2_O	CH_4_	Total GWP (kg CO_2_ eq)
1. Diesel (L)	569.73	0.10	0.10	602.78
2. N Fertilizer (kg)	7171.93	0.12	0.17	7212.07
3. Phosphate (P_2_O_5_) (kg)	1313.32	0.02	0.02	1318.99
4. Potassium (K_2_O) (kg)	95.67	0.00	0.00	96.64
5. Electricity (kW h)	16,731.19	0.18	0.18	16,788.16
Total GHG (kg)	25,881.83	0.42	0.46	
Total GWP (kg CO_2_ eq)	25,881.83	125.22	11.59	26,018.64

**Fig. 1:**
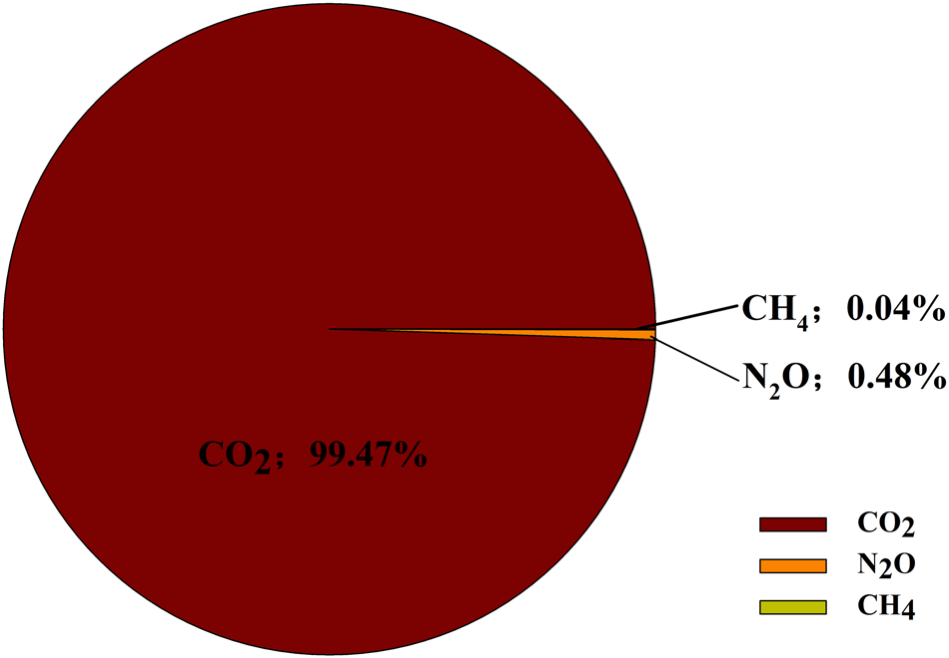
The share of CO_2_, N_2_O, and CH_4_ of GWP in wolfberry production systems

**Fig. 2:**
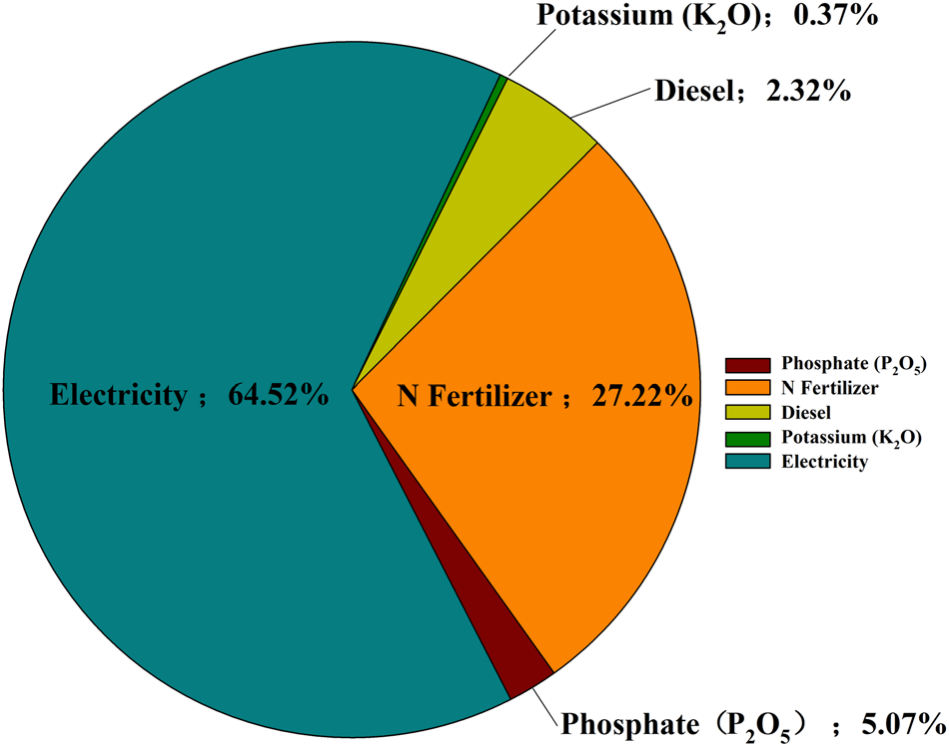
Share of GWP of different inputs in wolfberry production

The total GWP in the wolfberry planting system was 26,018.64 kg CO_2_ eq ha^−1^ and the shares of CO_2_, N_2_O, and CH_4_ were 99.47%, 0.48%, and negligible, respectively, with CO_2_ being dominant in terms of the greenhouse effect (Fig. 1). The share of GWP of different inputs was 64.52% for electricity consumption, 27.72% for N fertilizer, 5.07% for phosphate fertilizer, 2.32% for diesel, and 0.37% for potassium fertilizer, respectively (Fig. 2). The highest share was highly correlated with electricity consumption for irrigation, followed by N fertilizer input. The emission of CO_2_ contributed overwhelmingly to the total GWP of the GHG emissions with both N_2_O and CH_4_ being rather small. Bakhtiari et al. (2015) revealed electricity had the highest share of GHG emissions in potato production. And the greatest share of GWP was also found being related to electricity consumption in an irrigated wheat cultivation system (Yousefi et al. 2015) as well as in cucumber production (Khoshnevisan et al. 2013) while fertilizer application holds the highest portion in the total GHG emissions in oil olive cultivation (Rajaeifar et al. 2014) and soybean farming (Mohammadi et al. 2013).

In the wolfberry production system, the production of wolfberry fruits would cause GWP generation of 5.78 kg CO_2_ eq kg^−1^, 2.6 kg CO_2_ eq m^−2^, 0.08 kg CO_2_ eq MJ^−1^ by input energy, or 0.16 kg CO_2_eq MJ^−1^ of energy output. The production of 1 kg of almonds generates 1.5 kg CO_2_ eq emissions in California, the USA (Kendall et al. 2015). Pergola et al (2013) reported that the GWP of conventional and organic lemon as well as orange production were 0.12, 0.04, 0.13, and 0.04 kg CO_2_ eq kg^−1^, respectively, in Sicily, Italy. GWPs for organic and conventional orange production on small farms (<75 ha) are 0.084 and 0.112 CO_2_ eq kg^−1^, respectively, in Brazil (Knudsen et al. 2011). GHG emissions for truly efficient and inefficient orange orchards are 0.075, 0.0939, and 0.126 kg CO_2_ eq m^−2^, respectively, in Iran (Nabavi-Pelesaraei et al. 2014) while that for apple production system is 0.26 kg CO_2_ eq m^−2^ in Switzerland (Mouron et al. 2006). In addition, Yousefi et al (2015) reported GWP generation of 1.67 kg kg^−1^, 1.17 kg m^−2^, and 0.19 kg CO_2_ eq MJ^−1^ of input energy in irrigated wheat production systems and 0.37 kg kg^−1^, 0.07 kg m^−2^, and 0.05 kg CO_2_ eq MJ^−1^ by input energy in rain-fed wheat production as well. Sugar beet production has a GWP generation of 0.024 ton CO_2_ eq ton^−1^ clean beets harvested in the UK, while it has been estimated to be between 0.174 and 0.093 ton CO_2_ eq ton^−1^ winter wheat grain in Europe (Tzilivakis et al. 2005). Clearly, the wolfberry production system is not efficient in the use of energy and resources.

### Pathways for Improving Energy use and Abating GHG Emissions

The threat of climate change has called for the reorientation of development direction. Low carbon agriculture is one of the key sectors to achieve transformation towards low carbon growth and the shift to low carbon farming is a critical step in this connection.

From a policy perspective, innovative policy strategies should be formulated to underpin green growth initiatives. First, smallholder farmland transfer (concentration) should be encouraged through cooperatives, companies, and family farms for scale production. Large farms (>5 ha) uses less chemical fertilizer and consume lower energy for irrigation while the total energy output is higher compared with small farms (<1 ha) and medium farms (1–5 ha) (Pishgar-Komleh et al. 2012). Second, credit (small credit and low interest credit), tax breaks, and subsidies are needed to encourage the shift to low carbon farming.

Efficient transfer of knowledge to farmers through innovative extension systems with the combination of top-down and bottom-up pathways should be carried out and research deliver robust and cost-effective technologies; nonetheless farmers’ involvement in them is particularly important.

A seasonal gradient water pricing system, consisting of a basic quota price based on the crop water requirement for the growing season as well as an escalating pricing mechanism for the nongrowing season, should be in place, to leverage substantial water saving.

Greater priority should be given to irrigation for GHG emissions reduction. Irrigation is a carbon-intensive operation. Batty and Keller (1980) reported that energy required for surface irrigation was 3184 (MJ ha^−1^) for 0 m lift, 56,250 (MJ ha^−1^) for 50 m lift and 109,317 (MJ ha^−1^) for 100 m lift. Increasing irrigation efficiency is vital in reducing GHG emissions and raising energy productivity in wolfberry production in the Jingtai region. Currently extravagant water use for irrigation leads to a lot of water wasted and in turn high electricity consumption for lifting water from the Yellow River. Low irrigation water use efficiency results from inefficient irrigation methods (flooding), high irrigation quotas, and an irrational water pricing mechanism (Wang et al. 2012). New irrigation techniques, for instance, small tube, drip, subsurface drip, etc. should be encouraged by precision technological extension and incentives. Moreover, the use of crop residue and gravel mulching provides another alternative to reduce evaporation from the soil surface, thus, raising water use efficiency and potentially increasing wolfberry yields (Zeng et al. 2013).

For agro-chemicals, synthetic fertilizers in particular nitrogenous fertilizer are a principal source of CO_2_ and N_2_O emissions (Lal 2004). Further, embodied fossil fuel carbon associated with nitrogen fertilizer accounts for one of the largest energy inputs to agriculture. The chemical fertilizer use rate in Gansu is close to that of developed countries, while the effective utilization rate is about 30% (Gao 2008). Hence nitrogen fertilizer is a top priority target for GHG reduction. Efforts should be directed to enhance nitrogen fertilizer use efficiency, reducing reliance on chemical fertilizers, and optimizing application rates without negatively affecting productivity and soil fertility. Fertilizer application based on soil nutrient diagnosis, precision placement, and appropriate timing of fertilization (for example, through fertigation by modern irrigation technology), farm manure, N-fixing legume crops, biogas residue, etc. are recommended.

## Conclusions

In the wolfberry systems considered in this study, the largest share of energy inputs was electricity consumption (68.52%), related to lifting water for irrigation, followed by fertilizer use (11.37%) and chemicals (5.45%). Energy ratio was 0.52 with inclusion of pruning wood and the energy productivity was low (0.014 kg MJ^−1^). Direct energy inputs were much greater than indirect energy consumption and nonrenewable energy use far larger than the renewable energy input.

Total GHG emissions were 25,882.72 kg ha^−1^ with CO_2_ being overwhelming. And total GWP was 26,018.64 kg CO_2_ eq ha^−1^ with the highest share coming from electricity consumption for irrigation. The emission of CO_2_ contributed most to the GWP.

The production system highly depends on nonrenewable energy (88.52%) associated with electricity consumption for irrigation, fertilization, and biocide use and these operations are C intensive, intensifying GHG emissions.

Irrigation consumes a large amount of energy due to backward irrigation methods, mainly flood irrigation and broader border irrigation. Furthermore the water pricing system leads high irrigation quotas, as a consequent, contributing to increased GHG emissions.

A range of options can be employed to reduce the rate of nonrenewable energy use and mitigate environmental burdens, including conversion to low carbon farming, decreasing nonrenewable energy inputs, and increasing performance of nonrenewable energy inputs. Policy initiatives, including smallholder farmland transfer (concentration) for scale production, credits, tax breaks, and subsidies are strongly recommended to underpin GHG reductions. Efficient transfer of knowledge to farmers and robust and cost-effective technologies formulated by research are essential as well. Innovative water pricing systems, improvement of irrigation efficiency by the adoption of new techniques and optimized irrigation norms are crucial. In addition, greater priority should be given to judicious use of chemical fertilizers and biocides with particular attention to reducing the use of synthetic N fertilizers.
